# Supplementary material for: Lipopolysaccharide-Enhanced Responses against Aryl Hydrocarbon Receptor in FcgRIIb-Deficient Macrophages, a Profound Impact of an Environmental Toxin on a Lupus-Like Mouse Model
Source: Int J Mol Sci. 2021 Apr 18;22(8):4199. doi: 10.3390/ijms22084199 (PMC8073880; doi:10.3390/ijms22084199)
Supplement: Supplementary file 1 [file ijms-22-04199-s001.zip › ijms-1123937-supplementary.pdf]

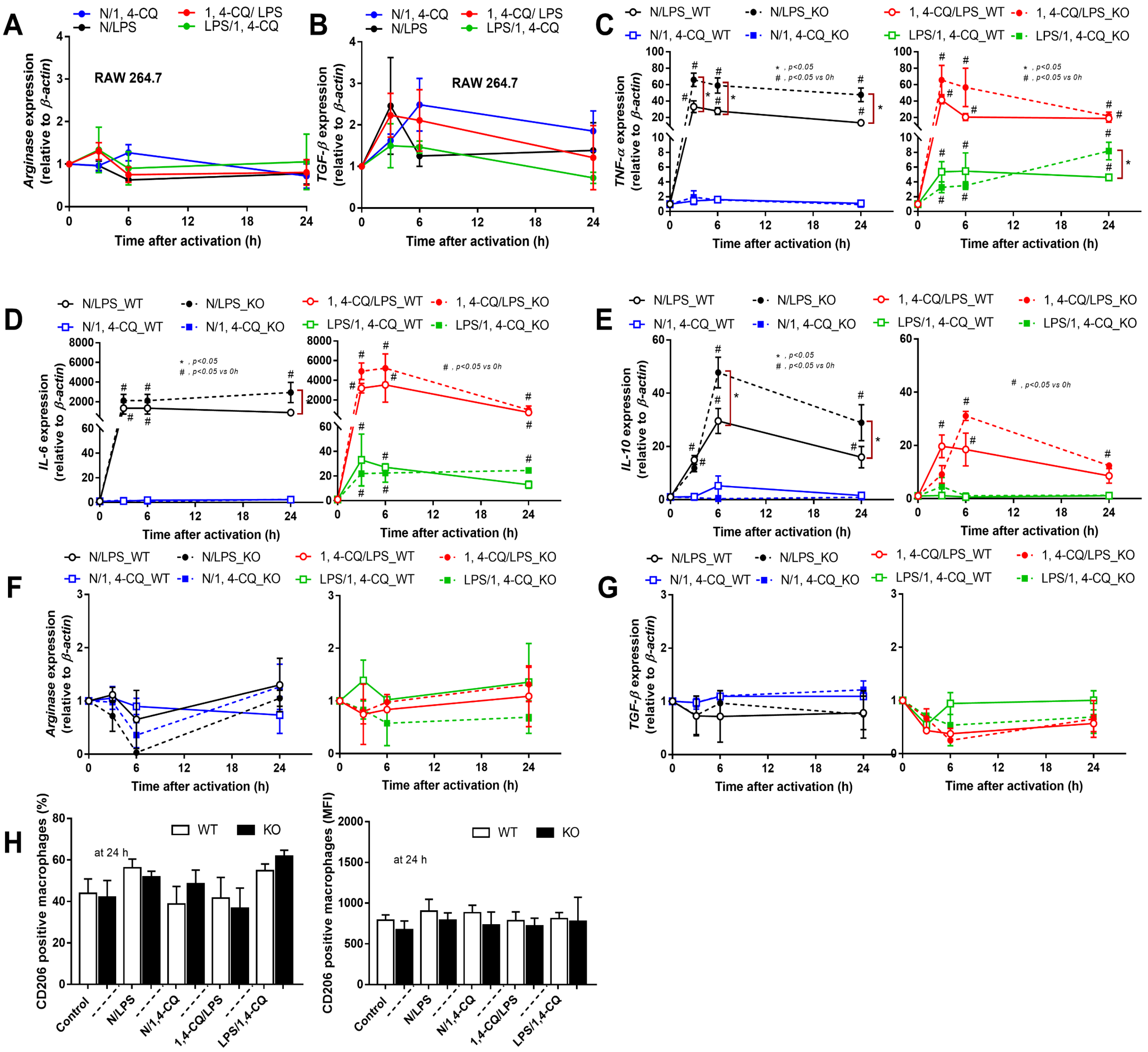


**Supplement Fig 1.** The characteristic responses of RAW246.7 cells against a single activation by an aryl hydrocarbon receptor activator; 1,4-chrysenequinone (N/1,4-CQ), or lipopolysaccharide (N/LPS) (a positive-control stimulator) or the activation with the pre-treatment protocol (1,4-CQ/LPS and LPS/1,4-CQ) as determined by M2 macrophage polarization (gene expression of *arginase* and *TGF-β*) (A, B) are demonstrated. Additoinally, the responses of wild type (WT) and FcgRIIb -/- (KO) macrophages as indicated by gene expression of cytokines (C, D) and M2 polarization (*arginase* and *TGF-β*) together with flow cytometry analysis of CD206 at 24 h post-stimulation (D) are demonstrated (experiments had been done in triplicate).

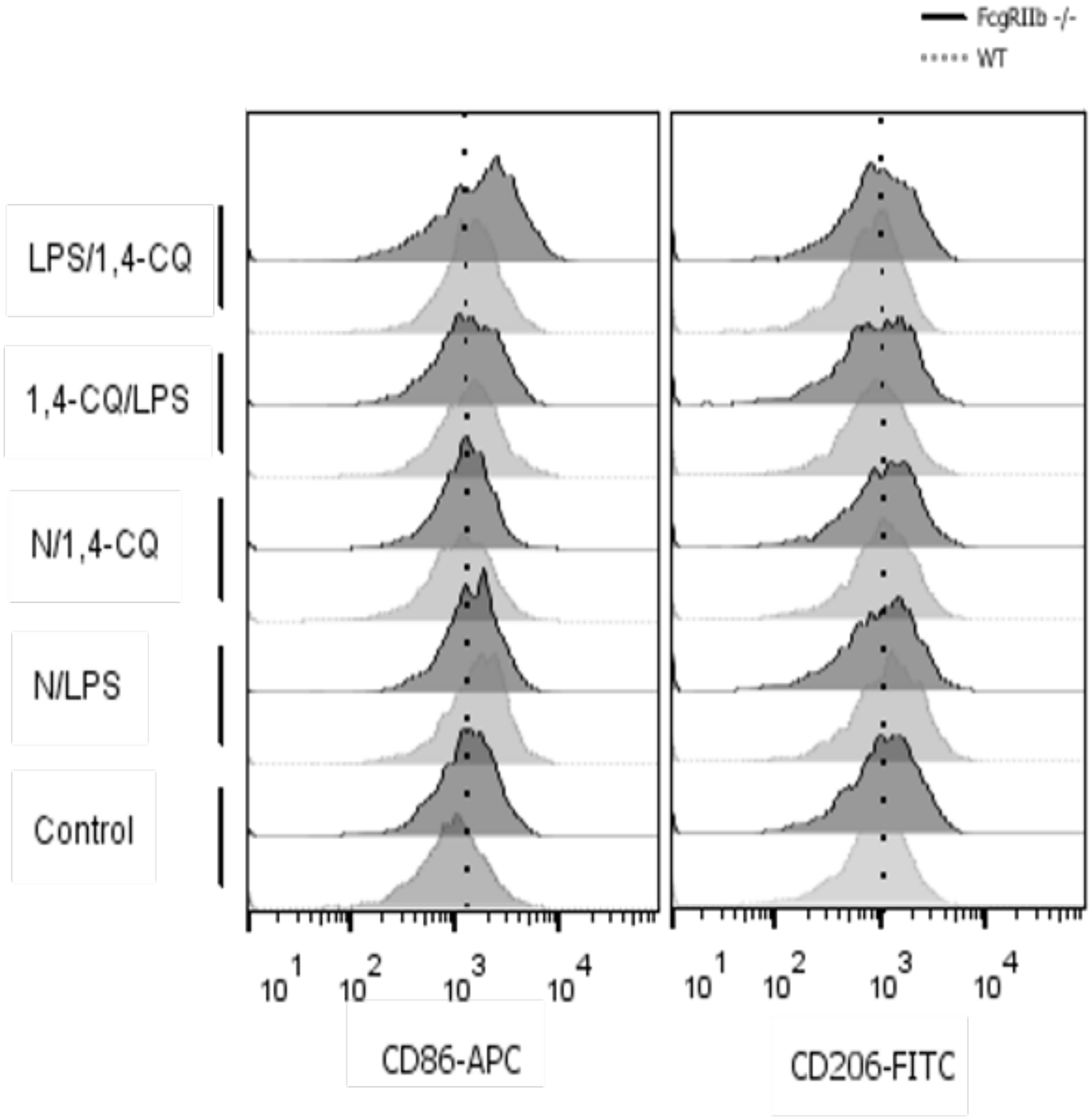


**Supplement Fig 2.** The representative pictures of flow cytometry analysis of CD86 and CD206, a marker of M1 and M2 macrophage polarization, respectively, at 24 h post-stimulation by an aryl hydrocarbon receptor activator, 1,4-chrysenequinone (N/1,4-CQ), or a positive-control stimulator, lipopolysaccharide (N/LPS) and the activation after the pre-treatment protocol (1,4-CQ/LPS and LPS/1,4-CQ) or control media alone (control) are demonstrated.
